# Structure–Function Relationships of the Repeat Domains of RTX Toxins

**DOI:** 10.3390/toxins11110657

**Published:** 2019-11-12

**Authors:** Ulrich Baumann

**Affiliations:** Institute of Biochemistry, University of Cologne, Zülpicherstrasse 47, D-50674 Cologne, Germany; ubaumann@uni-koeln.de; Tel.: +49-221-470-3208

**Keywords:** RTX toxin, type I secretion, calcium, internal chaperone, protein folding, tertiary structure

## Abstract

RTX proteins are a large family of polypeptides of mainly Gram-negative origin that are secreted into the extracellular medium by a type I secretion system featuring a non-cleavable C-terminal secretion signal, which is preceded by a variable number of nine-residue tandem repeats. The three-dimensional structure forms a parallel β-roll, where β-strands of two parallel sheets are connected by calcium-binding linkers in such a way that a right-handed spiral is built. The Ca^2+^ ions are an integral part of the structure, which cannot form without them. The structural determinants of this unique architecture will be reviewed with its conservations and variations together with the implication for secretion and folding of these proteins. The general purpose of the RTX domains appears to act as an internal chaperone that keeps the polypeptide unfolded in the calcium-deprived cytosol and triggers folding in the calcium-rich extracellular medium. A rather recent addition to the structural biology of the RTX toxin is a variant occurring in a large RTX adhesin, where this non-canonical β-roll binds to ice and diatoms.

## 1. Introduction

The repeats-in-toxins (RTX) family represents a collection of proteins from mainly Gram-negative bacterial origin [1,2,3,4]. These polypeptides vastly vary in size including molecular weights from about 10 kDa to larger than 1 MDa. Functional diversity is present as well: While many members of this family are cytolytic toxins, e.g., *Escherichia coli* hemolysin HlyA or *Bordetella pertussis* adenylate cyclase CyaA, other members are the nodulation-signaling protein NodO protein from *Rhizobacterium leguminosarum,* or possess hydrolytic activity, e.g., proteases such as the serralysins from *Pseudomonas aeruginosa* or lipases like LipA from *Serratia marcescens* (reviewed in [5]). A common denominator of probably all of these proteins is the secretion by a type I secretion system (T1SS), which requires a C-terminal non-cleavable signal sequence that is generally preceded by a variable number of nonapeptide tandem repeats [6,7,8,9,10]. These repeats coined the denotation of the RTX toxins [3]. 

The T1SS machinery itself consists of an inner-membrane ABC transporter that recognizes the C-terminal secretion signal of the passenger protein, a membrane fusion protein spanning the periplasmic space, and an outer membrane pore protein. The three components are thought to assemble into a continuous exit passage upon engagement of the ABC transporter with the secretion signal. Therefore, the traditional view is that secretion occurs without periplasmic intermediates, but there are at least some exceptions to this [11]. Owing to the narrow structure of the outer membrane protein tunnel, as seen paradigmatically in the crystal structure of TolC, secretion most likely requires an unfolded or only partially folded passenger protein. The necessity for passage in the unfolded state was for the first time experimentally proven for HasA, maybe the only example for a protein being secreted by a type I system not possessing the characteristic RTX nonapeptide motifs (reviewed in [8,12]). On the other hand, HasA secretion is dependent on the chaperone SecB, contrary to the RTX proteins of which the secretion is chaperone-independent. SecB’s engagement with HasA delays its folding by an order of magnitude, which is necessary considering that the C-terminal location of the translocation signal requires complete synthesis of the polypeptide chain and the very fast folding of HasA.

The nonapeptide repeats, which coin the denotation of the RTX proteins, possess the consensus sequence ^1^XUXGGXGXD^9^, where U stands for a non-polar residue, e.g., leucine. These repeats were early shown to bind Ca^2+^ ions [13]. There is a positive correlation of the number of repeats with the molecular weight of the protein, thus varying the number of nonapeptides from about 4 to more than 50. Larger number of repeats usually come in blocks of about six to eight, where they are arranged in a tandem fashion. These blocks are separated by linkers of variable length and amino acid sequence. Deviations from the consensus sequence mentioned above occur mainly at the edges of the blocks where the structural constraints are less strict.

The functional significance of the repeat domain (RD or RTX domain) has been less clear for some time. For example, the repeats of *E. coli* hemolysin HlyA have early on been implied in binding to receptors of erythrocytes [13], but it was also clear that many RTX proteins would not bind to erythrocytes. The calcium-binding property has also been implicated in the formation of cation pore channels, which again is not a feature of all RTX-containing proteins.

Beside the RTX repeats, another conspicuous structural feature of all these proteins is the rare occurrence of cysteine residues, with most proteins containing either none cysteines or just one cysteine. In other words, there is a lack of disulfide bonds, which are otherwise quite frequent in extracellular proteins.

In analogy to the role of SecB in the secretion of HasA, one could assume that the RTX domains also delay folding of the respective proteins and that folding is triggered by the extracellular binding of calcium ions, of which the concentration is sub-micromolar in the cytosol, but millimolar in the extracellular medium. 

The requirements of the actual translocation process for the RTX repeats and concomitantly for calcium ions has been less clear for some time. While a number of studies agreed that the RTX repeats are not the secretion signal, Létoffé and Wandersman [14] showed that the RTX repeats are not involved in recognition by the T1SS but are needed for the efficient translocation of larger passenger proteins. 

In this review, the focus will be on the structural features of the RTX domain and its implications for folding and secretion of the polypeptide chain.

## 2. RTX Repeats Form a Unique Parallel β-Roll Domain Which Contains Ca^2+^ Ions as an Integral Part

The first three-dimensional structure of a protein containing RTX repeats was that of the alkaline protease AprA from *Ps. aeruginosa* (PDB entry 1KAP) [15], which features five RTX repeats in its C-terminal region. Other experimental structures with RTX domains that are available in the PDB are cited in Table 1.

AprA is a 50 kDa metalloprotease that is secreted by a type I secretion system into the extracellular medium. It is homologous to the 50 kDa protease from *Serratia marcescens* (serralysin) and the metalloproteases PrtA, PrtB, PrtC, and PrtG from *Erwinia chrysanthemi*, where the latter have been also extensively been studied with respect to their secretion mechanism [34,35,36]. All these proteases belong to the serralysin family [37], which is annotated as family M10B in the MEROPS protease data base [38]. Many or all of them are encoded on an operon together with the necessary components for secretion, i.e., for alkaline protease the ABC transporter aprD, the membrane fusion protein aprE, and the outer membrane protein aprF, followed by the protease-encoding aprA gene [39]. Some of the serralyins producing organisms also carry a gene for an inhibitor of the protease, which is located at the end of the operon. The inhibitors are small 10 kDa proteins that are located in the periplasm of the bacteria, although type I secretion usually occurs in one step without periplasmic intermediates [16,19,40]. As with the other proteins secreted by the type I system, serralysins contain a C-terminal non-cleavable secretion signal, which is preceded by five copies of the characteristic 9-residue sequence motif ^1^XUXGGXGXD^9^.

**Figure 1:**
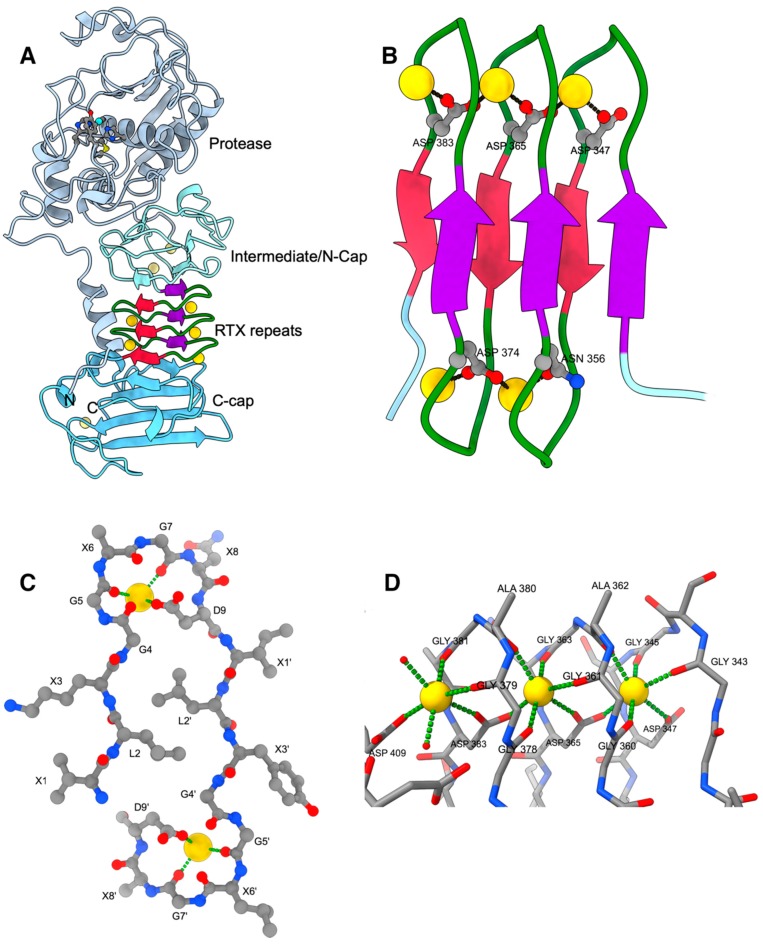
(**A**) Cartoon of AprA (PDB entry 1KAP). Ca^2+^ ions are shown as golden spheres. The N-terminal helix and the protease domain are depicted in grey with the active-site Zn^2+^ as cyan sphere together with the residues mentioned in the text. The β-roll is depicted in crimson and purple with green calcium-binding loops. The intermediate/N-cap and C-cap subdomains are depicted in different shades of light blue. (**B**) Close-up of the parallel β-roll. (**C**) Side-view of one turn of the parallel β-roll depicting 2 tandem nonapeptides of sequence XLXGGXGXD with X being variable amino acids. (**D**) Details of the octahedral Ca^2+^ coordination by the GGXGXD turn. The left Ca^2+^ ion is the most accessible one and the only that can be exchanged by Pb^2+^, for example. Its coordination sphere is completed by water molecules depicted as small red spheres. All figures were prepared with ChimeraX [42].

Serralysins possess an N-terminal metzincin-type protease domain (Figure 1A) [41], which harbors the characteristic HEXXHXXGXXHP metzincin metalloprotease sequence motif with the ultimate proline being characteristic for the serralysins. The three histidines in this motif are zinc ligands and the glutamic acid serves as catalytic base. About 30 residues downstream of this motif resides the S-[T/V/L/I]-MSYW met-turn finger print, where the name-coining methionine of the metzincins is located, together with a tyrosine residue that functions as electrophilic agent stabilizing the tetrahedral transition state of the peptide bond hydrolysis reaction.

After the protease domain there is an extended β-sheet architecture, which can be formally subdivided into three parts: an intermediate/N-cap domain, the repeat domain containing the RTX motif, and a C-terminal domain, which caps the RTX domain and where the secretion signal is located within the last ~20 amino acids. Most important in the context of RTX toxins is the middle part, which was termed parallel β-roll, sometimes also called a parallel β-helix, although the latter refers originally to a different architecture. The parallel β-roll is built up by the succession of the RTX nonapeptide repeats together with bound calcium ions. Here, the first three residues of a nonapeptide ^1^XUXGGXGXD^9^ form a short beta strand, while the following six amino acids build a turn that binds to calcium ions and connects this nonapetide to the following one downstream in the sequence. Further continuation of this leads to a right-handed spiral of β-strands with calcium ions bridging the aspartic acid residues at positions n and n + 18 (Figure 1B,C). This results in a packing of two parallel β-sheets against each other with opposite strand directions. The characteristic β-sheet main-chain hydrogen bonds are formed only within the two sheets and not between them, i.e., the arrangement is rather a sandwich of two β-sheets than a β-barrel. The loops on top and bottom of the β-sandwich wrap around ’pearls-on-a-string’ aligned calcium ions, where neighbouring Ca^2+^ ions are bridged by the Asp-9 carboxylate group of a particular repeat motif.

This architecture differs drastically from the parallel β-helix discovered at about the same time in the structure of pectate lyase C from *Erwinia chrysanthemi*, which does not contain repeating sequence motifs and which is built up by three β-sheets and which does not require metal ions for stability [43,44].

The requirements for the individual amino acids in the nine-residue repeats became well understood from the three-dimensional structure. Residues 1 to 3 XUX form a short, non-twisted β-strand with the sidechain of the second residue ‘U’ pointing into the interior of β-roll. Leucine residues are preferred because they nicely interdigitate forming a tight hydrophobic core of the sandwich, but other hydrophobic residues—e.g., valine or phenylalanine—are found as well. The sidechains of the X-residues point to the exterior of the β-roll, and residues with a high propensity for β-strands are most frequent at this position; for example, threonine residues are often located at the first position. The following six residues GGXGXD build two calcium-binding half sites, where the carbonyl oxygens of the Gly-5 and Gly-7 together with the *anti* lone pair of one oxygen of the carboxylate group of Asp-9 form half of the octahedral coordination sphere of a calcium ion (Figure 1D). The remaining three ligands completing calcium ion coordination originate from a nonapeptide motif located 18 residues further downstream in the sequence (i.e., the residues involved are located in the over the next motif). These ligands are the *syn* lone pair of an aspartic acid carboxylate group oxygen atom Asp-9’ and the carbonyl oxygens of residues Gly4’ and X6’. 

An analysis of the main chain dihedral angles revealed that the conformation of the amino acids at positions 5 and 7 are energetically unfavorable for non-glycine residues due to their positive phi-angles (‘Ramachandran disallowed’). Residue 4, which is also preferably a glycine, adopts a conformation with main-chain dihedral angles of Φ/Ψ ≈ −70/−30, a combination which is accessible for all amino acids. However, the presence of a sidechain as small as a methyl group would lead to clashes with the Ca^2+^-coordinating carboxylate group of the aspartate residue at position 6. 

It is very noteworthy that the parallel β-roll domain mediates contacts to the intermediate domain, on which the protease module resides, to the N-terminal helix, which connects in the tertiary structure the C-terminal end part of the structure with the protease domain, and to the C-cap structure located at the C-terminal end of the primary structure. Thus, structuring of the parallel β-roll during the folding process would definitely impact the folding of the entire molecule.

Beside the calcium ions bound to the RD, there are three additional Ca^2+^ sites, of which two are in the intermediate/N-Cap domain. Their purpose might be to confer additional stability to the tertiary structure, which as most RTX proteins lacks disulphide bonds, although these two sites in the intermediate domain share some structural similarity to those bound to the RTX repeats.

## 3. RTX Domains Containing more than One Block of Repeats Build a Compact Assembly that Probably Serves as a Folding Nucleus for the Functional Passenger Domains

**Figure 2:**
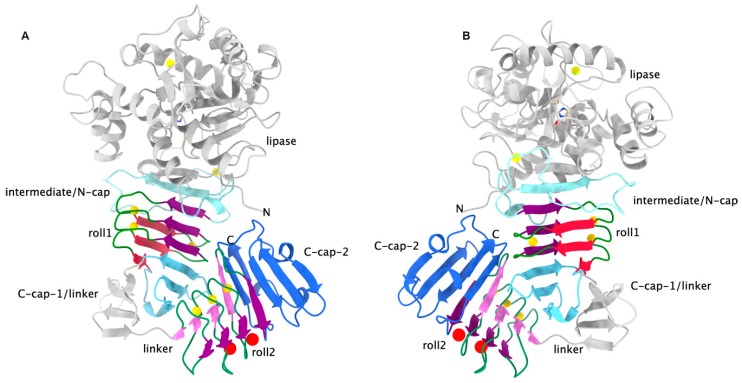
Two views on LipA rotated by about 180 deg. around the vertical (PDB entry 2QUA). (**A**): The color coding is as in Figure 1. This time, there are two C-caps, one for each RTX β-roll. Water molecules occupying one edge of the second roll are shown as red spheres. Roll 2 has only at one side calcium ions bound. (**B**): Same as in A but rotated by about 180 degrees around the vertical. The interaction between the two β-roll domains is clearly visible.

Initially, we thought that polypeptides with a greater number of repeat units simply had a larger β-roll, i.e., that the additional repeat units were just added directly to the existing structure like adding additional turns to an α-helix. However, closer inspection revealed that the nonapeptide repeats come in blocks of about six to eight and that these blocks are separated by linker with sequences very different from the consensus sequence described above. So far, there are only two structures available in the PDB data bank that feature multiple blocks of repeats (i.e., two of them), namely the extracellular lipase LipA from *Serratia marcescens* [28] (Figure 2) and of a related extracellular lipase from *Pseudomonas* sp. [29]. Besides the significant deviations of the RTX consensus sequence of the individual repeats, a major finding here was that the two RDs are not flexibly connected but interact strongly with each other. Furthermore, especially in the second block, the deviations from the consensus sequence are significant. For example, there are insertions of one residue and the crucial aspartic acid residue is substituted in every other repeat, leading to only one side of that β-roll being occupied by Ca^2+^ ions, while at the corresponding positions at the other edge the density has been interpreted as water molecules. The very similar *Pseudomonas* sp. MIS38 lipase also exhibits variations from the consensus sequence but has two calcium ions bound at that side of the β-roll as well [29].

The tight association of the two β-roll domains and both their interactions with the lipase domain led to the reanimation of the proposal for an internal chaperone function of the RTX repeats, an idea which had emerged already with the first crystal structure of AprA. The purpose of it would be to keep the polypeptide chain in an unfolded state in the cytosol, where the Ca^2+^ concentration is low, and to induce folding of the RDs and concomitantly of the whole protein extracellularly by the higher amounts of Ca^2+^ present there [28].

## 4. Ca^2+^-Induced Folding of RTX Domains

The Ca^2+^ ions bound by the RDs were recognized as integral part of the 3D structure of the folded RTX domains from the beginning on when the crystal structure of alkaline protease had been determined. The metal ions are buried in the structure. Only the calcium ions located at the edge of the parallel β-roll structure are partially solvent-accessible and are coordinated in part by water molecules; only these ions can be removed by EDTA under non-denaturing conditions or be substituted for example by Pb^2+^ ions (in Figure 1D this is the upper left Ca^2+^ ion for example). Thus, complete removal of calcium ions from folded β-rolls requires—in addition to chelators—a denaturing agent, e.g., urea. The calcium ions bridge spatially adjacent aspartate residues that are 18 amino acids apart in the primary structure. The distance between the centers of adjacent carboxylate groups is only about 4.8 Å, like the distance between the calcium ions. Consequently, in the absence of calcium ions, the electrostatic repulsion of the carboxylate groups will make the folded structure unstable.

This was first demonstrated by folding experiments employing an artificially designed β-roll consisting of the chemically synthesized peptide H_2_N-WLS-[GGSGNDNLS]_8_-COOH, which contains eight exact repeats, and revealed in the absence of Ca^2+^ ions a random coil structure [45]. Addition of Ca^2+^ together with PEG8000 led to conformational changes and a CD spectrum consistent with the formation of a mainly β-sheet structure. In accordance with the crucial bridging of spatially adjacent carboxylate groups from the aspartic acid residues at position 9 of the consensus motif, it turned out that—for this artificial peptide—the presence of Ca^2+^ in concentrations of about 1 mM was essential for folding. Other cations such as Mg^2+^ or Tb^3+^ could not substitute for calcium, revealing an interesting specificity of the cation binding sites, especially considering the rather high intracellular Mg^2+^ concentrations (about 100 mM although most of it is bound), which are then incompetent to induce folding.

However, this artificially designed β-roll was only folding in the presence of rather high concentrations of PEG8000, an unspecific stabilizer of tertiary structure by molecular crowding. Similar observations were made using ‘repeat-domain-only’ fragments of *Bordetella pertussis* CyaA, where a polypeptide consisting solely of nine repeats (residues 1528-1612 or 1529-1612) did not bind to Ca^2+^ [46,47], while in another study an increase in Ca^2+^-affinity by molecular crowding of the CyaA RD(1530-1680) by Ficoll was observed [48]. For the synthetic β-roll described above, the lability of the folded state may have at least in part arisen from a sub-optimally designed sequence with an asparagine residue at position 7, which should be replaced by other residues such as threonine. 

However, there is another reason why peptides only comprising the RTX nonapeptides are less prone to fold spontaneously in the presence of Ca^2+^. Interestingly, the folding kinetics of the synthetic β-roll revealed a lag phase, which depended on the peptide concentration, as did the folding of the β-roll. Finally, the appearance of polymers and insoluble fibrous aggregates was observed. This led to a model where cooperative folding is initiated by a few already structured monomers, of which the formation is overall rate-limiting. In accordance with this assumption, the lag phase and consequently overall folding could be accelerated by adding a small amount of pre-formed beta roll (‘seeding’). It was furthermore assumed that structure-induction and polymerization leading to fibrous insoluble aggregates occurred via edge-to-edge aggregation of the β-roll units, where an edge of the beta sheets of a folded monomer or oligomer interacts with unfolded molecules, thus providing a β-sheet nucleation face. Since the edges of a folded monomer are unprotected, edge-to-edge aggregation will occur, leading to large insoluble polymers resembling amyloid fibrils.

Indeed, other studies showed that a protection especially of the C-terminal edge of the β-roll is necessary for calcium-induced folding, as it is generally needed for avoiding aggregation of β-sheet structures. So it was early observed that the β-helix structure of pectate lyase employs a capping of the edges by loops and helices in order to avoid edge-to-edge aggregation [49]. This is exactly what apparently happens in the RTX domains, too; in Figure 1 and Figure 2 these capping elements are denoted as N-cap/intermediate/linker and C-cap. The need for the C-terminally flanking regions of an RTX block was recognized during studies of the repeat domain of *Bordetella pertussis* CyaA, where it was found that a fragment including residues 1529–1681 corresponding to the most C-terminal RTX block could be stably expressed and refolded when the residues of the C-terminal segment were included. The crystal structure of this fragment revealed a typical beta roll structure where the C-terminal side is covered by additional beta strands and an alpha helix [27,46]. Another study showed that even replacement of the C-terminal cap by YFP was sufficient for folding, although much higher Ca^2+^ concentrations were required [47].

The Ca^2+^-induced folding of RTX domains which were flanked by additional segments has been in-depth characterized on various constructs of the RD of adenylate cyclase CyaA from *Bordetella pertussis* [47,48,50,51,52,53,54,55] as well as for example on the alkaline protease AprA from *Ps. aeruginosa* [56]. All these studies confirmed that folding requires calcium concentrations in the range of about 0.1 to 0.2 mM, which is about 1000 times higher than that present in the cytosol. In the absence of Ca^2+^, all constructs exhibited random-coil characteristics while at the same time staying soluble—i.e., no aggregation was observed—owing to the highly hydrophilic nature of the RTX repeats.

All these results strongly hint at an unusual internal chaperone function of the RTX domains. They keep the polypeptide chains in the calcium-depleted cytosolic medium in a (mainly) unfolded state, which is required for secretion. Translocation to the extracellular medium then occurs in the unfolded state from the C-terminus, which bears the secretion signal, to the N-terminus. The exposure of the C-terminal region containing RTX repeats and adjacent segments to the high Ca^2+^ concentrations in the extracellular medium result in folding of the β-roll, which aids in structuring the whole polypeptide chain. This disorder-to-order transition was shown to be essential for secretion of the CyaA toxin from *B. pertussis* [52]. The vectorial C-to-N folding has been demonstrated in a study by Bumba et al. [27], who also could thereby show that this mechanism leads to a more efficient secretion by a ‘Brownian ratchet’, since the folded domains of the polypeptide chains are unable to slide back through the secretion tunnel. This consolidates the long-standing issue whether the RTX repeats are needed for secretion by type I secretion systems: small and/or unfolded domains can be fused to the C-terminal secretion signal without the intervening RTX repeats, while larger passengers will remain unfolded if fused to a large enough RTX domain, or, like HasA, will require chaperones that keep them in the unfolded state in the cytosol. The requirement for Ca^2+^ ions for secretion (in contrast to folding) has been recently examined in some detail as well with conflicting results. While one publication reported a HlyA secretion rate independent of the calcium concentration [57], another study showed a strong increase [27]. 

## 5. Structure of RTX Adhesins Reveals the Presence of Canonical and Non-Canonical RTX Repeats

A very interesting addition to the structural repertoire of the RTX domains is represented by a hyperactive antifreeze protein *Mp*IBP (IBP for ice binding protein) from the Gram-negative Antarctic bacterium *Marinomonas primoryensis*. The protein belongs to the RTX adhesins and has a molecular weight of about 1.5 MDa. It shows a very strong depression of the freezing point of water by interaction with lattice planes of hexagonal ice, but also anchors the bacterium to diatoms. The protein is retained in the outer membrane and possesses a very repetitive structure which is shared by other RTX adhesins and multifunctional autoprocessing RTX toxins (MARTX, [58]). In the context of the RTX repeat domains most interesting are here the so-called region-IV and region-V (RIV and RV), which contain the RTX repeats and the T1SS sequence. The structure of these two domains as well as of the other pieces have been determined [32,33,59]. 

RV is at the very C-terminal end and comprises four copies of the RTX-nonapeptide plus the C-cap including the secretion signal. Four Ca^2+^ ions are bound to the loops.

**Figure 3:**
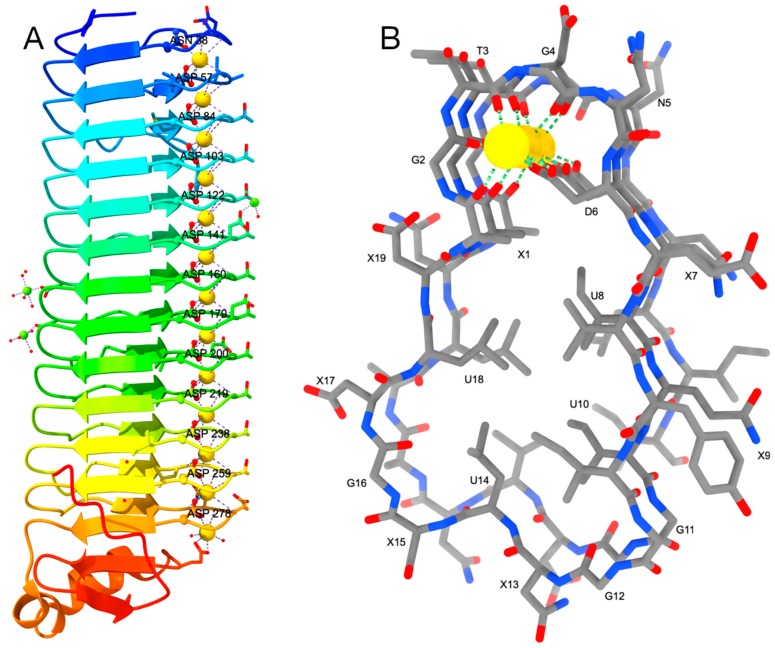
(**A**) the parallel β-roll of the *Mp*IBP is colored as rainbow from N-terminus (blue) to C-terminus (red). Oxygen atoms are painted in red, Ca^2+^ and Mg^2+^ ions are shown as golden and green spheres, respectively. Waters are shown as small red spheres. (**B**) Side-view of the roll depicting the motif XGTGND-XUXU-GGXUXG-XUX where U stands for a non-polar and X for a polar amino acid. Carbon atoms are shown in grey, nitrogen and oxygen in blue and red, respectively, and Ca^2+^ ions as golden spheres.

RIV, which is very closely located in the sequence upstream of RV, is the ice-binding domain of *Mp*IBP and possesses an atypical β-roll where Ca^2+^ ions are only bound to one side of the protein (Figure 3). One turn of this new β-roll comprises 19 amino acids instead of 18, i.e. 2 × 9, in the canonical structure. The repeat sequence deviates from the canonical (XUXGGXGXD)_2_ motif and reads now XGTGND-XUXU-GGXUXG-XUX, where the first six residues build the Ca^2+^-binding turn like in the canonical structure, followed by a four-residue β-strand. The second six-residue loop has a very different sequence and consequently does not bind to Ca^2+^ ions, which therefore are all located at the other edge of the roll. Further differences are that the two β-sheets of the sandwich differ in length: one is four residues long, the other three, although small variations occur. Similar deviations were observed in some parts of the second roll domain of *S. marcescens* lipase (see above). However, in the *Mp*IBP these are rather the rule than the exception. The bound Ca^2+^ ions are described as not hexa-coordinated in an octahedral geometry but approximately hepta-coordinated. The aspartic acid from the second half site is providing both *syn* lone pairs of the two carboxylate oxygen atoms, although one of the oxygen atoms is more distant (~3.0 Å instead of 2.3 Å). Furthermore, all 12 19-residue repeats form one continuous β-roll and are not divided into smaller blocks.

Since the aspartate residues bridge the calcium ions and they are deeply buried in the interior of the loops, it is very likely that also this segment adopts a random coil structure in the cytosol and folds in the extracellular medium. Thus, this domain may have a twofold role: aiding secretion, and binding to ice and diatoms.

## 6. Conclusions and Outlook

The RTX-domains is a calcium-triggered switch that keeps the polypeptides in a secretion-competent unfolded state in the cytosol and induces folding of the translocated proteins in the external, calcium-rich medium. Keeping the polypeptide unfolded and therefore dysfunctional in the cytosol may also mitigate possible toxic effects. It must be stressed that the tight binding of calcium ions is not reflected by a dissociation constant since the binding is accompanied by folding and concomitant burial of the ions and is therefore irreversible. Folding and formation of Ca^2+^ sites is highly cooperative, and it requires Ca^2+^ in a concentration of at least 100 µM. The calcium binding sites can be degenerated with large deviations from the canonical (XUXGGXGXD)_2_ motif leading to an asymmetric distribution of the ions. The RTX domain also aids secretion, at least for larger passengers. While this appears to be common for all RTX proteins, the example of the antifreeze protein shows that these domains could also possess further functions, e.g., binding to ice surfaces or receptors; for CyaA the integrin receptor binding site has been mapped to the RTX domain [60]. The structures of complete toxins are not available yet, however owing to the advances in single particle cryo-EM they may be well under way. They would provide more insight into the arrangement of the RTX blocks and may help to discover additional functions. The specific calcium-induced folding of RTX domains has also raised interest in biotechnological applications [61,62].

## Figures and Tables

**Table 1:** RTX protein structure in the PDB (www.rcsb.org)

Protein	PDB Entry	No of Repeats XUXGGXGXD
*Ps. aeruginosa* alkaline protease *APRA_PSEAE*	1KAP [15], 1JIW [16], 1AKL [17], 3VI1	5
*S. marcescens* metalloprotease *PRZN_SERMA*	1SAT [18], 1SMP [19], 1AF0, 4I35, 5D7W [20]	5
*S. marcescens* sp. Metalloprotease *PRTZN_SERME*	1SRP [21]	5
*Erwinia chrysanthemi* metalloprotease PrtC *PRTC_DICCH*	1GO8 [22], 1K7G [23], 1K7I, 1K7Q, 3HB2 [24], 3HBU, 3HBV, 3HDA	5
*Caulobacter vibroides* S-Layer protein *A0A0H3C8J1_CAUVN*	5N97, 5N8P [25]	4
*Azotobacter vinelandii* mannrinan-C5-Epimerase *ALGE4_AZOVI*	2AGM, 2ML1, 2ML2, 2ML3 [26]	6
*Bordetella pertussis* adenlyate cyclase *CYAA_BORP1*	5CVW, 5CXL [27]	8
*Serratia marcescens* lipase LipA *Q5933_SERMA*	2QUA, 2QUB [28]	5 + 8
*Ps. Fluorescence* extracellular lipase *Q9RBY1_9PSED*	2Z8X, 2Z8Z, 2ZJ6, 2ZJ7, 2ZVD, 3A6Z, 3A70 [29]	5 + 8
*Pseudomonas* sp. psychrophilic metalloprotease *O69771_9PSED*	1H71 [30], 1G9K, 1O0Q, 1O0T, 1OM6, 1OM7, 1OM8, 1OMJ	5
*Flaviobacterium* sp. alkaline metalloprotease *DOVMS8_9FLAO*	3U1R [31]	5
*Marinomonas primoryensis* ice-binding protein region IV *A1YIY3_9GAMM*	3P4G [32]	12 19-residue repeats
*Marinomonas primoryensis ice-binding protein* region *V A1YIY3_9GAMM*	5 JUH [33]	4
